# Prevalence and correlates of infertility related psychological stress in women with infertility: a cross-sectional hospital based survey

**DOI:** 10.1186/s40359-022-00804-w

**Published:** 2022-04-07

**Authors:** Ashebir Getachew Teklemicheal, Eyasu Mesfin Kassa, Eskinder Kebede Weldetensaye

**Affiliations:** 1 Gandhi Memorial Hospital, Addis Ababa, Ethiopia; 2 Department of Obstetrics and Gynecology, School of Medicine, Addis Ababa University, Addis Ababa, Ethiopia

**Keywords:** Infertility, Psychological stress, Personal stress, Marital stress, Social stress

## Abstract

**Background:**

Psychological stress is commonly found among infertile women. Untreated stress can affect negatively the success of infertility treatment. Most of the available knowledge is generated from developed countries and is largely based on women undergoing In vitro fertilization (IVF) treatment. However, very little is known on infertile women from Ethiopia including other countries in East Africa. The aim of this study was to determine the prevalence of psychological stress and possible demographic-clinical correlates in Ethiopian women suffering from infertility.

**Method:**

This cross sectional descriptive correlation study was conducted from May to August, 2021 involving 96 women undergoing a non-IVF treatment for infertility at Gandhi Memorial and Tikur Anbessa hospitals. The sampling was continuous and based on inclusion criteria that include infertility duration of a minimum one year, female sex and Ethiopian nationality. Data on Infertility related stress was collected using the Copenhagen Multi‐Centre Psychosocial Infertility-Fertility Problem Stress questionner administered by an interviewer. The socio demographic and clinical factors were collected using Amharic version of structured interviewer administered questionnaire and chart review. The analysis of relationship between infertility related stress and background variables was done with an independent sample t-test or one-way ANOVA statistics supplemented with effect size assessment.

**Results:**

The prevalence of infertility related stress was overall 92.71% (95% CI, 87–98%). The personal, marital, and social subdomain mean scores (SD) were 2.74 (.80), 1.54 (.81) and 1.90 (.80), respectively. Infertility related stress was higher for those women who were: aged above 35, living in a cohabitation marital type, has no living children, and with 4–6 years duration of infertility (all *P* < 0.05). Whereas, there was insufficient evidence to suggest that infertility related stress varies by education, income, knowledge of cause of infertility or history of past treatment (all *P* > 0.05).

**Conclusions:**

The prevalence of psychological stress among Ethiopia women having infertility was very high. The results provide preliminary evidence that infertility related stress is associated with age, marital status, motherhood status and duration of infertility. Responsible bodies need to avail psychological screening and services prioritizing women at higher risk of developing stress.

## Background

infertility is a failure to establish clinical pregnancy after one year of unprotected sexual intercourse [1]. In 2010, sub-Saharan Africa had the second highest prevalence of primary infertility, and the 4th highest prevalence of secondary infertility of seven world regions studied [2]. In Ethiopia, the prevalence of primary infertility ranges from 1.4 to 9.0% whereas the prevalence of secondary infertility ranges from 2.5 to 15.1% [3]. Infertility can lead to psychological stress that may arise from personal stress, marital relationships stress & social networks pressure [4]. According to personality theories failure to achieve the goal of parenthood, a favorite personal identity, is believed to be the root cause for psychological distress [5, 6].

The reported prevalence of infertility related stress is quite variable and ranges from 20 to 93% [7–9]. There is a general consensus among researchers that there are sub-group of women (estimated to be 20–25%) who develop a moderate to severe degree of stress, which is pathological as it may lead to mental health problems [10].

A number of factors have been studied as possible reasons for variability of stress scores. Commonly studied resource related variables education and income revealed inconsistent results with some studies showing association while others showed no association with infertility related stress [11–13]. According to [6] a blocked identity or role will result in more distress for those with few alternate identities. Of all role related variables studied the most consistently reported to have an effect is motherhood role, with loss of motherhood role (childnessness) consistently related to higher level of stress [13]. Many researchers reported that infertility related stress is higher among women who are undergoing infertility treatment [12–14]. However, the question of the extent to which distress among infertile women is due to the condition of infertility itself or to infertility treatment remains unanswered [15, 16].

Cognizant of the high prevalence and serious health consequences of infertility related stress, the European Society Human Reproduction Endocrinology recommend for establishing a mechanism for identification and provision of psychological care by infertility clinic staff [17]. Information on magnitude of infertility related stress is needed to help evidence based policy decisions for availing psychological help services. However, in Ethiopia very little is known on infertility related stress, and although use of information’s obtained from elsewhere for policy making might be possible the available evidences have limitations: firstly, most of studies are done in a socio-cultural setting characterized by white, educated and high income women which is very different from ours; Secondly, nearly all studies are done among women undergoing IVF treatment which is different from a non-IVF treatment clinic in terms of patient’s psychological burden. This study aims to determine the magnitude of infertility related psychological stress and explore for associated factors among Ethiopian infertile women seeking treatment.

## Methods

### Study design

A descriptive correlational study design was used – to study self-reported psychological stress in women with infertility seen between May and August, 2021 at public hospitals.

### Study setting

The study was conducted in two of the three public infertility clinics located in Addis Ababa, Ethiopia, a developing country located in East Africa. While the infertility clinics are located in the capital city, infertile women come to this clinic, not only from the capital of Ethiopia, but also from all places of Ethiopia, urban and rural. The diagnostic tests available include: pregnancy test, hormonal assessment (follicle stimulation hormone, luteinizing hormone, progesterone, estrogen, prolactin, thyroid stimulation hormone, T4), ultrasound examination, hysterosalpingography and hysteroscopy. Treatment services provided include: treatment of ovulatory dysfunction, hysteroscopy resection of endometrial adhesions and removal of polyps, laparoscopic surgery for correcting tubal and peritoneal problems, and open surgeries for myomectomy. The staff of both clinics includes Reproductive Endocrinology & Infertility specialists and clinical nurses.

### Subjects

All women who were seen at the Infertility units of study hospitals during the study period were approached and invited to participate in the study at the end of their clinical session, and recruitment continued until the required sample size of 96 was achieved. Enrolment was based on inclusion criteria that include infertility duration of a minimum one year and Ethiopian nationality.

Sample size for estimating prevalence of stress (primary objective) was calculated based on sample size determination for single proportion in survey type of studies [18]. Anticipating the prevalence of infertility stress in infertile females as 80% based on a study done in India [8], and taking a relative precision level of 0.08 calculated as 10% of the estimated prevalence, the minimum number of participants required for this study was estimated as 96. Using the G*Power 3 power analysis program [19], pre-hoc power analysis (for an alpha level of 0.05, two-sided testing) showed that the achieved sample size of 96 was sufficient to detect a moderate effect size of 0.58 in stress scores among compared groups with a power of 80% for two groups design. The effect size of 0.58 was taken from a meta-analysis study done to assess the effect sizes of psychosocial consequences of infertility in Iranian women [20]. The sample size of the current research subjects, 96, seems adequate since the uncertainty margin of + 8 percentage points we chose to tolerate in estimation of prevalence of infertility related psychological stress was within the acceptable level of error for medial researches. According to Conroy [21] for exploratory study, a margin of error of + 10% is acceptable. Likewise, the sample size achieved in this study seems suitable in detecting differences in psychological stress mean scores of moderate size between compared groups since the conventionally acceptable minimum value of power of a study (defined as the probability of finding the difference in two study groups if it actually exists) that is at least 80% was met.

### Measures

Data on infertility related stress was collected using the Copenhagen Multi‐Centre Psychosocial Infertility-Fertility Problem Stress Scales (COMPI-FPPS) developed by Schmidt in Denmark which is a widely used tool to measure the level of infertility-related stress[22]. We used the short version of the COMPI questionnaire that has 9 items with 3 items in each of the three subdomains of stress, namely: personal, marital and social stress [23]. Infertility related stress in the personal domain reflected the stress that infertility had produced on the person’s mental and physical health, Infertility related stress in the marital domain assessed the stress that infertility had produced on marital and sexual relationships, while Infertility-related stress in the social domain assessed the extent to which infertility had caused strain on social relations with friends, family and colleagues. Seven of the items are scored using a four-point Likert scale of severity ranging from (1. not at all, 2. a little, 3. some, and 4. a great deal), while two of the items (1 each from personal and social subdomains) are scored on a five-point Likert scale ranging from 1 (strongly disagree) to 5 (strongly agree) [24]. After getting written permission to use the COMPI-FPPS questionner from the developer we translated the English version in to Amharic language and conducted cultural adaptation as per the recommendations provided by Ortiz and Cruz [25]. The process completed include: (1) two forward translation was done by translators of which one was the Principal Investigator (PI), (2) Synthesis of the first version of questioner by merging works of both translators by the PI, (3) cross cultural linguistic validation (evaluation for semantic and conceptual equivalence) was done by expert panel comprising 5 members, (4) synthesis of the final version done, and (5) the final version was pilot tested on 10 cases. Cronbach’s alpha coefficient [26] for the total items was 0.76, which suggests the adequate reliability [27] of the Ethiopian Amharic language version of COMPI-FPPS questioner in measuring infertility related stress among Ethiopian women.

There are other tools useful for studying psychological stress among women diagnosed with infertility of which the Fertility Problem Inventory (FPI) and Fertility Quality of Life Tool (FertiQoL) are the most widely used [28]. The FPI originally developed by Newton in 1999 is a measure of the impact of fertility problems on five domains of life in women with infertility. The FPI has good internal consistency reliability (0.6 to 0.7) in measuring FPI scales, and an adequate construct validity [29]. The FPI’s reported reliability assessment score is comparable with our assessment of the COMPI-FPPS done using this study’s dataset (0.7). The one downside characteristics is high patient burden (46 items) compared to the COMPI-FPPS (9 items). The other commonly used tool FertiQoL is a measure of the impact of fertility problems and its treatment on quality of life. Evidence suggests good internal consistency reliability in measuring stress and adequate construct validity. the Cronbach’s reliability statistics reported by the developers were overall satisfactory, in the range of 0.72 and 0.92, which is comparable with this study’s finding of 0.76 [15]. The FertiQoL questioner has advantageous not present with COMPI-FPPS that include: can be used for assessment of the broader construct of quality of life and has a treatment section that can be used to separately analyze psychological effect due to infertility treatment from those due to childlessness, which is important given the different emotional challenges of each. One major downside is high patient burden (24 items) compared to the COMPI-FPPS (9 items).

Data on socio demographic and clinical factors were assessed using Amharic version of structured and semi structured interviewer administered questionnaire and chart review. The selection of variables included was informed by previous studies and theories relevant to infertility stress. Childlessness was measured by a question—do you have living children and was used as a measure of the role of motherhood fulfillment. Relationship type was measured as formally married (meaning having a legally or religiously certified marital status) and cohabitation. Employed (public, private, self) were grouped together for comparison with housewives. The type of infertility was determined by a question about history of previous pregnancy with a no or yes response indicating a primary and secondary infertility, respectively. Cases with known causes of infertility (male, female or mixed factor) were grouped together for comparison against unknown causes (unexplained and under investigation).

### Procedure

Data collectors explained the study, and individuals who gave an informed consent were interviewed by health care professionals trained in data collection. Women were interviewed in the absence of their partner and in a private environment. At the end of an interview session all study participants advised about the availability of a psychological help service that can be accessed in case they wish to seek the service.

### Statistical analysis

We did the Shapiro–Wilk test to assess whether our data follows the normal distribution by inputting the combined stress mean score observed. The test statistics produced a P-value of 0.14 which being greater than an alpha level of 0.05 indicated that the data tested is normally distributed and parametric tests can be used.

The overall prevalence of infertility related stress were computed by first calculating individual subjects average scores for responses to all 9 stress questioner items followed by calculating the proportion of participants whose combined infertility related mean score is above 1. The stress mean scores for the combined and sub domains (personal, marital & social) were derived by calculating the average score for responses to all and domain relevant questions, respectively. The t-test for unpaired samples or one-way ANOVA statistics followed as appropriate by a Boferroni post-hoc test was computed to explore whether infertility related stress scores (combined as well as subdomain scores) differ by background characteristics groups. To quantify the magnitude of differences between compared groups mean stresses scores the Cohen d effect size statistics for two independent groups was computed and Cohen d values of 0.2, 0.5 and 0.8 were interpreted as small, medium, and large differences, respectively [30, 31]. For all statistical tests, P-values < 0.05 (two-sided testing) were considered statistically significant. All data were analyzed by Stata 10 statistical software packages for data analysis.

## Results

### Demographic and clinical characteristics of study participants

All of the 96 women approached to participate in the study agreed. The mean age of participants was 31.5 (SD = 5.9) years and the majority of women (58.3%) belonged to the 26–30 (33.3%) & 31–35 (25%) age categories. Most (85%) of the women received some form of formal education with a third of them educated at tertiary level. Study participants mean household income was 7135 (SD = 5767) Ethiopian Birr. The majority of participants were unemployed house-wives (68.8%). The majority of women (92.7%) were married formally, while the rest few (7.3%) were in a co-habitation type of relationship. The majority of women (4 out of 5) were childless, thus did not achieved the role of a motherhood status. The mean duration of infertility was 4.2 (SD = 3.8) years; only 50 of the women were classifiable as per the standard clinical cause classification, while the rest 46 were not classifiable as their diagnostic work-up was not completed at the time of the data collection; a history of past treatment was found in a third of the sample with all except one reported a non-IVF treatment. Table 1 summarizes study participants characteristics.

**Table 1:** Socio-demographic and clinical characteristics of study participants (*N* = 96)

Characteristics	Number	Percent
*Age (years)*		
Mean ± SD (range)^a^		31.5 ± 5.9 (20–48)
20–25	16	16.7
26–30	32	33.3
31–35	24	25.0
36–40	18	18.7
41–48	6	6.3
*Education*		
No formal education	15	15.6
Primary	25	26.1
Secondary	24	25.0
Tertiary	32	33.3
*Household monthly income*^*b*^		
Mean + SD (range)		7135 ± 5767 (2000–25,000)
≤ 5000	41	42.7
> 5000	55	57.3
*Employment status*		
Employed	30	31.2
Housewife	66	68.8
*Relationship type*		
Married	89	92.7
Cohabiting	7	7.3
*Living child*		
Yes	21	21.9
No	75	78.1
*Infertility type*		
Primary	45	46.8
Secondary	51	53.1
*Infertility duration (years)*		
Mean + SD (range)		4.2 ± 3.8 (1–17)
1–3	43	44.7
4–7	46	47.9
≥ 8	7	7.2
*Cause of infertility*		
Female	46	47.9
Male	2	2.1
Combined	2	2.1
Unexplained	0	0
Unclassified	46	47.9
*Past infertility treatment*		
Received treatment		
Yes	30	31.2
No	66	68.8
Type of treatment received		
IVF	1	3.4
Non-IVF	29	96.6

a = standard deviation, b = Ethiopian birr

### Levels of infertility related stress

The overall prevalence of infertility related stress among participants was 92.71% (95% CI, 87–98%), with 47% reported a moderate to severe degree of stress. The data shown in Table 2 overall suggests that compared to the marital and social sub-domains the personal sub-domain contributed much to participants’ infertility related stress.

**Table 2:** Frequency relative percentage score and mean score for each of infertility related stress subdomains (N = 96)

Stress domains	Frequency of women with mean score above 1^a^		Relative percentage score (%)^b^	Mean score (SD)	95% CI
	n	%			
Personal stress	89	92.7	44.3	2.74 (.80)	2.58–2.90
Marital stress	46	47.9	24.9	1.54 (.81)	1.38–1.71
Social stress	71	73.9	30.7	1.90 (.80)	1.74–2.07

a: subdomain items specific mean score of above one which is the cut-off score for stress; b: Relative scores were computed by dividing the mean score for each scale by the sum of all mean item scores (e.g., Relative percentage for personal stress = mean score (personal stress) / Sum of all mean scores)

### Socio-demographic variables relationship with infertility related stress

The combined, personal and marital infertility related stress scores were higher for women older than 35 year of age compared to those who were younger, for those living in a co-habitation relationship compared to formally married (all *P* < 0.05; *d* = 0.52–1.01). Those women who don’t have living children compared to those who have children were more stressed in the combined, personal, and social stress (all *P* < 0.05: *d* = 0.49–0.56). On the other hand, there was insufficient evidence to suggest that infertility related stress varies by employment status or household income (all *P* > 0.05).

### Clinical variables relationship with infertility related stress

A one-way ANOVA revealed a difference in the combined (*P* = 0.02) and social (*P* = 0.01) stress scores among women with the 1 to 3, 4 to 7 and above 8 years duration of infertility, a Post hoc analysis with Boferroni adjustment indicated that the combined and social stress mean scores were higher in women with duration of infertility of 4–7 years compared to those with 1–3 years (all *P* < 0.05) (Table 4). Table 3 shows that there was insufficient evidence to suggest that infertility related stress varies by knowledge of cause of infertility or past treatment history (all *P* > 0.05). The combined infertility related stress mean score for women treated previously with ovulation stimulation drugs (n = 24), IVF (n = 1) and surgery (n = 9) was 2.1, 2.1, and 1.9, respectively (Table 4).

**Table 3:** Comparison of infertility related stress mean scores based on socio-demographic and clinical characteristics groups (*N* = 96)

Characteristics	Combined stress			Personal stress			Marital stress			Social stress		
	Mean (SD)	*t*	*d*	Mean (SD)	*t*	*d*	Mean (SD)	*t*	*d*	Mean (SD)	*t*	*d*
*Age in years*												
< 35	1.97 (.58)	2.4*	0.52	2.60 (.76)	2.5*	0.54	1.40 (.74)	2.5*	0.56	1.89 (.80)	0.2	0.06
≥ 35	2.28 (.58)			3.04 (.82)			1.85 (.90)			1.94 (.81)		
*Employment*												
Housewives	2.10 (.67)	0.3	0.08	2.68 (.75)	0.4	0.11	1.57 (.75)	0.2	0.05	2.04 (.96)	1.1	0.22
Employed	2.05 (.56)			2.77 (.75)			1,53 (.75)			1.84 (.72)		
*Income*												
≤ 5000	2.03 (.54)	0.6	0.13	2.71 (.79)	0.4	0.09	1.39 (.67)	1.9	0.40	1.96 (.84)	0.7	0.15
> 5000	2.11 (.65)			2.78 (.83)			1.71 (.93)			1.84 (.76)		
*Relationship type*												
Married	2.02 (.58)	2.6*	1.01	2.69 (.80)	2.3*	0.90	1.50 (.79)	2.02*	0.78	1.88 (.81)	1.2	0.49
Cohabitation	2.62 (.38)			3.42 (.56)			2.14 (.99)			2.28 (.67)		
*Living children*												
Yes	1.84 (.75)	2.01*	0.49	2.39 (.91)	2.2*	0.56	1.55 (1.02)	0.04	.01	1.57 (.77)	2.2*	0.53
No	2.13 (.53)			2.84 (.75)			1.54 (.75)			2.00 (.79)		
*Cause*												
Known	2.05 (.61)	0.2	0.05	2.72 (.87)	0.2	0.05	1.56 (.81)	0.1	0.04	1.88 (.80)	0.3	0.07
Unknown	2.08 (.57)			2.76 (.74)			1.53 (.83)			1.94 (.81)		
*Past treatment*												
Yes	2.07 (.63)	0.1	0.02	2.82 (.92)	0.6	0.15	1.56 (.80)	0.1	0.02	1.85 (.79)	0.4	0.10
No	2.06 (.58)			2.70 (.75)			1.54 (.83)			1.93 (.81)		

t = independent sample t-test, d = Cohen’s effect size

* = p value for t-test < 0.05

**Table 4:** Comparison of infertility related stress mean score between duration of infertility groups (*N* = 96)

Stress domains	1–3 years		4–7 years		≥ 8 years		*F*(2, 93)^d^	Post hoc Bonferroni
	*Mean*	*SD*	*Mean*	*SD*	*Mean*	*SD*		
Combined stress	1.89^a^	.538	2.23^b^	.614	2.09^c^	.599	3.74*	a VS. b
Personal stress	2.58	.888	2.88	.716	2.85	.813	1.61	na^e^
Marital stress	1.44	.755	1.65	.877	1.47	.835	0.70	na
Social stress	1.65^a^	.577	2.14^b^	.928	1.95^c^	.755	4.49*	a VS. b

* = p value for F test < 0.05; Post hoc column shows which groups (a vs. b, a vs. c, b vs. c) are significantly different from one another, d = One-way ANOVA; e = not applicable

## Discussion

This study’s finding of a very high prevalence of infertility related stress is in line with study findings reported from developing countries for example studies from Cameroon [32], India [8], and Nigeria [33] reported a prevalence of 84.6%, 80%, and 87.2%, respectively. On the other hand our finding was higher by nearly threefold compared to results reported from developed countries 30.8% from Sweden [34]. Overall, this study finding of high prevalence of stress in Ethiopian women was not unexpected as the Ethiopian socio-cultural environment gives a prominent social status for women with children—for example it is customary to referee women with their children’s name as a way of respect.

The higher rank order for personal stress sub-domain found in this study was similar to findings from Greece, Germany, Croatia, Sweden and Hungary, but, our finding was different compared to what was found in china where social subdomain stand out, and Denmark where relational sub-domain ranks first [23].

Similar to our study finding several qualitative studies from Africa indicated that infertility affects the psychosocial and marital aspects of women’s lives. In Mozambique infertile women are excluded from important social ceremonies and fear divorce [35]. A study conducted among Malian women suffering from infertility found that infertile women lived with marital tensions, criticism from relatives, and stigmatization from the community [36]. Infertile women in Ghana report facing severe social stigma, marital strain and a range of mental health difficulties [37]. A survey done in Rwanda a threat to union dissolutions and sexual dysfunction were reported more frequently by infertile than fertile couples, and pressure by the in-laws is almost exclusively exercised by the family of the man [38]. A quantitative study done in Ghana reported that infertility has psychological, marital and social consequences on individuals as well as couples [39].

Our finding of more stress in the older women than younger women was similar to what many other researchers has found out [16, 40]. A lower stress in those younger might be due to better personal resources which is better in the younger compared to the older age group which according to resources theory might buffer the level of stress through better appraisal and control of stress. Yet, our finding contradicts with that of others [41, 42].

This study’s’ finding of higher stress among women in a co-habitation relationship compared to those who are married is consistent with one previous report from USA [43]. We think the higher stress among co-habitating women might be due to fear (or feeling insecure) of losing their relationship which is potentially easier compared to a legally established marriage. This study’s’ finding is in support of the security theory—Amir et al. [44] reported that less secure individuals reported more stress and secure individuals less distress.

Motherhood role deprivation (or not having a biological child) was found by this researcher to be related to higher stress which was consistent with what was previously reported from America [29, 45] and from Ghana [11]. Notably, similar to previous studies [13, 46, 47] our results revealed that motherhood role deprivation has a higher magnitude effect size on infertility related stress. This study finding is in support of the personality theory that states failure to achieve the goal of parenthood, a favorite personal identity, is the root cause for psychological distress [5, 6].

## Strengths and limitations

The important contributions of this study include: (1) to the best of our knowledge this study is first for Ethiopia including East Africa, (2) the study have provided rare evidence that infertility related stress is not moderated by a non-IVF treatment, and (3) this study is among the few that studied the effect of non-formal marital relationship (or cohabitation) on infertility related stress, and (4) our study adds to few studies that demonstrated the feasibility of using quantitative measure in studding infertility related stress in Africa.

This study’s main limitation is that clinic based study sample might not be representative of non-treatment seeking infertile population. However, a population based study reported previously from America [13] has shown no difference in stress scores between those seeking and not seeking treatment gives us a confidence that the treat to external validity might not be big. Further this study finding may not be readily generalizable to women who are enrolled in IVF treatment. It is also worth noting that the psychological stress results observed in the unclassified cause group could have been different if it was possible to classify them into one of the cause categories, namely: female, male, combined or unexplained. For example: a study done to assess the effect of a gender-specific infertility diagnosis on the psychological responses has showed women with a diagnosed female infertility experienced higher personal distress and high social stress than women experiencing a diagnosed male infertility [48]. Another study reported that women with combined-factor diagnosis reported significantly lower personal distress compared to women with other diagnosis [49].

## Conclusion

The prevalence of psychological stress among Ethiopia women with a diagnosis of infertility seeking care at non-IVF clinical centers was very high. Factors related with Infertility related stress were age, marital status, childnesness and duration of infertility. Responsible bodies need to avail psychological screening and services prioritizing women at higher risk of developing stress.

## Data Availability

The data underlying this article will be shared on reasonable request to the corresponding author.

## Abbreviations

COMPI-FPPS: Copenhagen Multi‐Centre Psychosocial Infertility-Fertility Problem Stress Scales
IVF: Invitro fertilization
